# Topology- and sequence-controlled β-amyloid recognition by a KLVFF/transferrin-functionalized csq porphyrinic zirconium MOF (PCN-222)

**DOI:** 10.3389/fchem.2026.1906393

**Published:** 2026-09-09

**Authors:** Xiao Cheng, Hongen Wei

**Affiliations:** 1 Department of Neurology, Shanxi Provincial People’s Hospital, Shanxi Medical University, Taiyuan, China; 2 Shanxi Key Laboratory of Neurobehavior and Brain Disease Control, Taiyuan, China

**Keywords:** Alzheimer’s disease, amyloid-β, csq-topology, KLVFF, molecular recognition, PCN-222, porphyrinic metal–organic framework, transferrin

## Abstract

Recognition of amyloid-β (Aβ) by structurally defined inorganic scaffolds offers a route to dissecting how framework geometry and surface chemistry govern peptide–material interactions. We report transferrin (Tf)/KLVFF-PCN-222, a bioinorganic recognition platform built by covalently co-grafting the Aβ-homologous pentapeptide KLVFF and transferrin (Tf) onto the csq-topology porphyrinic zirconium MOF PCN-222 via EDC/NHS chemistry. The one-dimensional hexagonal mesopore channels (∼3.7 nm) and the dense meso-tetrakis (4-carboxyphenyl)porphyrin (TCPP) sites of the csq framework provide geometric access for Aβ1–42 monomer diffusion and an array of peptide–porphyrin recognition contacts. Fluorescence quenching and isothermal titration calorimetry returned an apparent dissociation constant (*K*_d,app_) of 33.5 ± 4.2 nM for the Aβ1–42 monomer (apparent fluorescence-mode limit of detection (LOD) = 1.8 nM), and circular dichroism showed that the probe suppressed Aβ β-sheet conversion (14% vs. 41% β-sheet content at 24 h). A panel of structural controls—scrambled FFKLV, the narrow-pore fcu MOF UiO-66-NH_2_, and the TCPP-based ftw MOF-525—together with a selectivity screen against BSA, IgG, Tau, α-synuclein, and lysozyme (selectivity ratio >12 for Aβ1–42) indicates that KLVFF sequence specificity and csq mesopore geometry act cooperatively to drive recognition. The framework retains an intrinsic NIR photothermal property (photothermal conversion efficiency, η = 47.3 ± 1.8% at 808 nm); the photoacoustic measurement reported here is a probe concentration calibration only, and no Aβ-responsive photoacoustic signal was established. In an hCMEC/D3 *in vitro* monolayer, the apparent permeability coefficient (*P*_app_) reached 1.78 × 10^−5^ cm s^−1^ with ∼48% of the flux being TfR1-competition-sensitive, and SH-SY5Y viability was partially preserved in a tandem BBB–neuron Transwell model. All validations are *in vitro*. This work provides a topology-controlled, structure–function proof of concept for csq Zr-TCPP frameworks as bioinorganic platforms for Aβ recognition and conformational modulation.

## 1 Introduction

Alzheimer’s disease is, by almost every epidemiological metric, the defining neurological challenge of the coming decades. Forecasts from the Global Burden of Disease study place the worldwide dementia population at roughly 153 million by 2050, with AD accounting for 60%–80% of cases (GBD 2019 Dementia Forecasting Collaborators, 2022; Alzheimer’s Association, 2024)—a burden prominent enough to occupy the 2024 Lancet Standing Commission’s agenda alongside the WHO (Livingston et al., 2024). The amyloid cascade hypothesis frames pathological accumulation of amyloid-β (Aβ), particularly the Aβ42 isoform, as an early molecular event in AD pathogenesis that precedes clinical symptoms by 10–20 years (Hansson, 2021; Jack et al., 2024), which has made the Aβ monomer–oligomer–fibril axis a central target for molecular recognition studies. Existing clinical readouts of Aβ status are powerful but operationally demanding—CSF sampling requires invasive lumbar puncture (Hansson, 2021), and amyloid-PET depends on short-lived radiotracers and specialized infrastructure (Villemagne et al., 2018)—and, importantly for this study, they reveal little about how a chemically defined surface engages Aβ at the molecular level. Building structurally explicit *in vitro* systems that recognize Aβ therefore retains clear scientific value, primarily as a mechanistic lens on the peptide–material interface and on Aβ conformational behavior, rather than as a near-term diagnostic.

NIR photoacoustic imaging occupies an appealing position in biological sensing, combining the molecular contrast of optical absorption with the deep penetration of ultrasound to achieve centimeter-scale imaging at roughly 100 µm spatial resolution in tissue (Wang et al., 2016). Early demonstrations in AD rodent models have extended its reach to in-brain Aβ and cerebrovascular targets (Jiang and Pu, 2017; Zhai et al., 2024), and photothermal and photodynamic strategies for modulating Aβ aggregation have drawn related interest (Gao et al., 2025). What has attracted less systematic effort is the integration of NIR absorption with defined biomolecular recognition in a single, structurally characterized inorganic scaffold—one where framework geometry and recognition performance can be explicitly linked rather than empirically observed.

Porphyrinic MOFs offer a distinctive entry point here, combining intrinsic chromophore properties with the topological tunability of coordination chemistry (Lismont et al., 2017; Wang L. et al., 2018). Among Zr-meso-tetrakis (4-carboxyphenyl)porphyrin (TCPP) systems—assembled from Zr_6_O_4_(OH)_4_ clusters and TCPP—the structural diversity is remarkable: at least six crystallographic polymorphs have been identified depending on Zr_6_ connectivity and TCPP coordination mode, including csq PCN-222, she PCN-224, sqc PCN-225, shp PCN-223, scu NU-902, and ftw MOF-525 (Deria et al., 2016; Feng et al., 2012; Feng et al., 2014; Morris et al., 2012; Park et al., 2016). This diversity is not merely academic—topology directly determines pore geometry, site accessibility, and surface-site density, all of which plausibly influence how well a given framework can engage a macromolecular target such as Aβ. PCN-222 adopts the csq arrangement, pairing 8-connected [Zr_6_(µ3-O)4(µ3-OH)4] SBUs with 4-connected TCPP linkers to produce one-dimensional hexagonal mesopore channels (∼3.7 nm diameter, BET ∼2,200 m^2^ g^−1^) running along the c-axis (Feng et al., 2012). At 3.7 nm, these channels admit Aβ1–42 monomers (hydrodynamic radius ∼0.9 nm) and small oligomers, whereas the narrow fcu windows of UiO-66 (∼0.6 nm) would not. The TCPP chromophore contributes a strong Soret band at 400–450 nm and four Q-bands at 500–650 nm; their positions and intensities depend on metalation state, coordination environment, and peripheral substitution. Kafentzi et al. (2026) have reviewed the structure–electronics–function logic for porphyrinic MOF systems more broadly; specifically, PCN-222 has been applied in photodynamic therapy (Lu et al., 2014; Wang J. et al., 2022). Its potential as a platform for *in vitro* Aβ recognition and the structure-controlled questions this setting enables have received comparatively little attention. Beyond MOFs, a broad materials toolbox has been developed to recognize and modulate Aβ, and these classes differ in how recognition is achieved: organic photosensitizers and small-molecule dyes act largely by photo-oxygenating the peptide backbone; polymeric and micellar carriers work by shielding exposed hydrophobic surfaces; and metal, carbon, and quantum-dot nanomaterials exploit surface curvature and charge to divert the aggregation pathway. The trade-offs across these classes have recently been surveyed for the photothermal and the photodynamic modulation of Aβ aggregation (Gao et al., 2025). In most of these systems, however, the recognition event is inferred from the aggregation outcome rather than resolved at a defined interfacial geometry. Porphyrin-based systems are particularly relevant to the present design. Photoexcited meso-substituted porphyrins bind Aβ directly and suppress both its aggregation and its synaptic toxicity (Lee et al., 2015). This chemistry has been adapted into porphyrinic MOFs. For example, PCN-224 nanoparticles attenuate Aβ aggregation and neurotoxicity under near-infrared irradiation (Wang J. et al., 2018), while PCN-222 has been combined with indocyanine green and an RVG targeting peptide for photothermally enhanced photo-oxygenation (Wang J. et al., 2022). Closest to our own design are porphyrin–peptide nanoparticles. Liu et al. (2020) showed that a porphyrin conjugated to KLVFF—a pentapeptide whose C-terminal Phe–Phe pair is the diphenylalanine (FF) recognition motif excised from Aβ—self-assembles into nanoparticles that selectively engage Aβ and disassemble upon binding. More directly, Xia et al. (2025) assembled porphyrin-substituted phenylalanine–phenylalanine nanoparticles (TPP-FF NPs) from porphyrin-modified dipeptides and showed that they inhibit Aβ aggregation, disassemble preformed aggregates under illumination, suppress Cu^2+^-induced generation of reactive oxygen species, and act as quenchers for the sensitive fluorescence detection of Aβ oligomers. The FF motif is by itself sufficient to drive self-assembly into well-defined nanostructures (Reches and Gazit, 2003), and the same aromatic Phe–Phe and π–π contacts that underlie Aβ self-recognition are presented at high density by the TCPP struts lining the csq channels of PCN-222. This study therefore shares with TPP-FF NPs both the porphyrin recognition surface and the fluorescence quenching readout; the main difference lies in the scaffold. TPP-FF NPs are supramolecular self-assemblies, whereas PCN-222 is a crystalline framework in which pore geometry, site accessibility, and surface-site density are set by the underlying topology, which can therefore be varied systematically while the chromophore is held constant. What these porphyrin, porphyrin–peptide, and porphyrinic MOF systems establish is that a porphyrin surface can engage Aβ; what none of them addresses is whether the topology on which that surface is displayed is itself a design variable—the question the csq/fcu/ftw comparison reported here is built to answer.

On the molecular recognition side, two tools address distinct challenges. KLVFF—the pentapeptide matching Aβ residues 16–20—was first shown by Tjernberg et al. (1996) to arrest fibril formation through homosequence recognition with Aβ’s hydrophobic core, and it remains among the most studied Aβ self-recognition sequences (Zhang et al., 2013). Transferrin (Tf) engages TfR1, which is highly expressed on brain microvascular endothelium, to support receptor-mediated transcytosis across *in vitro* BBB models (Johnsen et al., 2019; Lajoie and Shusta, 2015); Tf-decorated nanocarriers have repeatedly demonstrated enhanced transcellular flux in these systems in previous studies (Pulgar, 2019; Wiley et al., 2013). Grafting both a sequence-specific Aβ recognition element and a Tf anchoring group onto a structurally defined inorganic scaffold—and asking how that dual design interacts with the underlying framework geometry—is the organizing question of this study.

We thus constructed Tf/KLVFF-PCN-222 by co-grafting the Aβ-recognition pentapeptide KLVFF and a Tf anchor onto csq-topology PCN-222 via EDC/NHS chemistry. We first examined how the framework recognizes Aβ1–42 and modulates its conformation and then assessed its apparent transcellular behavior in the hCMEC/D3 *in vitro* monolayer. The intrinsic NIR photothermal property of the porphyrinic framework is characterized as a material property rather than an Aβ-responsive readout. This work addresses three focal questions at the peptide–framework interface: whether KLVFF sequence specificity holds against a scrambled FFKLV control; how the csq-topology compares with the narrow-pore fcu framework (UiO-66-NH_2_) and the TCPP-based ftw framework (MOF-525) in engaging Aβ; and what mechanistic picture fluorescence quenching, ITC, CD, and computational docking–MD together support for the Aβ–porphyrin-MOF interface. All validations are *in vitro*; the photoacoustic measurement covers probe concentration calibration only, no Aβ-responsive photoacoustic signal was established, and clinical translation is not addressed here.

## 2 Materials and methods

### 2.1 Reagents and instruments

H_2_TCPP (≥97%), ZrOCl_2_·8H_2_O (≥99.5%), benzoic acid (≥99.5%), anhydrous DMF, human serum Holo-Tf (T0665), ThT, and HFIP were obtained from Sigma-Aldrich. EDC·HCl, NHS, and H2N-PEG4-NH2 were sourced from Thermo Fisher Scientific; Aβ1–42 (rPeptide A-1163-1), FITC-Aβ1–42 (AnaSpec AS-60479-01), and custom KLVFF-PEG4-NH2 (>95%, GL Biochem) were used as received. hCMEC/D3 (Millipore SCC066) and SH-SY5Y (ATCC CRL-2266) were obtained commercially. All solutions were prepared in Milli-Q water. The tissue-mimicking PA phantom used a commercially sourced defibrinated animal blood product or an equivalent hemoglobin-containing absorber; no fresh biological blood was collected for this study.

The following instruments were used in this study: Bruker D8 Advance (PXRD, Cu Kα, λ = 1.5418 Å); FEI Tecnai G2-F20 (TEM/EDS, 200 kV); Micromeritics ASAP 2020 (N_2_ sorption); Agilent 5800 (ICP-OES); Thermo Scientific ESCALAB 250Xi (XPS); Shimadzu UV-3600 Plus (UV–VIS–NIR); Jasco J-815 (CD); MicroCal PEAK-ITC; Malvern Zetasizer Nano ZS90; Hi-Tech MDL-III-808 laser (808 nm); FLIR A655sc (IR thermography); FUJIFILM VisualSonics Vevo LAZR-X (PA imaging); Leica TCS SP8 (confocal); and WPI EVOM2 (TEER).

### 2.2 Synthesis and activation of PCN-222

The synthesis was performed according to the method described by Feng et al. (2012), as outlined by Cavka et al. (2008). In brief, ZrOCl_2_·8H_2_O (70 mg, 0.217 mmol), H_2_TCPP (50 mg, 0.063 mmol), benzoic acid (2.7 g, 22.1 mmol; 350 mol equiv), and anhydrous DMF (8 mL) were added to a 25-mL sealed glass vial. Following sonication, the mixture was heated at 120 °C for 24 h. The deep-purple product was recovered by centrifugation at 4,000 rpm and subsequently washed three times with fresh DMF. HCl activation removed coordinated benzoic acid and residual Zr species by dispersing the crude solid in HCl/DMF (v/v = 1:99, 40 mL) at 120 °C for 12 h. Soxhlet exchange and supercritical CO_2_ drying were subsequently performed to preserve the ∼3.7-nm mesopore channels, which are vulnerable to capillary-force collapse under direct vacuum DMF removal: the activated material underwent continuous acetone reflux at 65 °C for 24 h, followed by six supercritical CO_2_ displacement cycles at 35 °C/10 MPa (2 h each, Tousimis Samdri-PVT-3D) before slow venting. The final PCN-222 was stored under argon. Phase purity was verified by matching the experimental PXRD pattern with the calculated data from CCDC 893545, along with evaluations of N_2_ sorption, TEM morphology, and ICP-OES Zr content.

### 2.3 Surface post-modification

Dual-peptide grafting proceeded in three EDC/NHS steps. Activated PCN-222 (20 mg) was dispersed in MES buffer (50 mM, pH 6.0, 10 mL); EDC·HCl (38 mg) and NHS (23 mg) were added, and the mixture was stirred at 25 °C for 30 min to activate surface carboxylates. After washing, the material was redispersed in PBS (pH 7.4) with H2N-PEG4-NH2 (1.32 mg) for 4 h, installing a bifunctional PEG4 linker (PCN-222-PEG4-NH2). Separately, Holo-Tf (5 mg) and KLVFF-PEG4-NH2 (0.39 mg; 1:8 M) were activated with EDC/NHS in MES for 15 min, combined with the PEG4-modified material, and stirred at 4 °C for 12 h. Three washes with 0.05% Tween-20/PBS, PBS, and ultrapure water preceded lyophilization. The same protocol—differing only in the conjugated components—was applied to all controls: Tf-PCN-222, KLVFF-PCN-222, scrFFKLV-PCN-222, Tf/KLVFF-UiO-66-NH_2_, and Tf/KLVFF-MOF-525. Tf loading was quantified by BCA assay (562 nm) after 0.1 M NaOH dissolution. Meanwhile, KLVFF loading was determined by reverse-phase HPLC (Agilent 1260, C18, 0.1% TFA gradient, 220 nm) after acid hydrolysis. Surface chemistry was verified using FTIR (amide I/II) and XPS N 1s.

### 2.4 Photophysical and photothermal characterization

Stability. Probe dispersions at 100 μg mL^−1^ in PBS, artificial CSF, and 10% FBS-PBS were stored at 4 °C for 7 days with DLS and UV–Vis monitoring every 24 h. Heating experiments. Probe dispersions (1.0 mL, 25–200 µg mL^−1^ in PBS) in quartz cuvettes were irradiated at 808 nm/1.0 W cm^−2^ for 600 s; cooling was tracked for a further 600 s. The photothermal conversion efficiency *η* was calculated by the Roper one-dimensional cooling method, as given in Equation 1 (Roper et al., 2007):

η is the photothermal conversion efficiency; *h* is the heat-transfer coefficient; *S* is the surface area of the sample container; Δ*T*_max_ is the maximum temperature increase of the dispersion at steady state; *Q*_dis_ is the baseline heat dissipated by the solvent and cuvette measured for probe-free PBS; *I* is the incident laser power density; *A*_808_ is the absorbance of the dispersion at 808 nm; *m* and *C*_p_ are the mass and specific heat capacity of the dispersion; τ_s_ is the system time constant obtained from the linear fit of the lnθ–*t* cooling curve; and θ is the dimensionless driving-force temperature.

$$\eta =\frac{hS\;\Delta T_{\max}-Q_{\text{dis}}}{I\left(1-10^{{-A}_{808}}\right)},$$ (1)

where *hS* = mCp/τs derives from the slope of the lnθ–t cooling curve. Photostability was assessed over five 600 s on–off irradiation cycles. PA calibration. In a tissue-mimicking phantom (1.0% Intralipid + 0.5% commercial defibrinated animal blood or equivalent), probe samples at 0–200 µg mL^−1^ were loaded into polyethylene tubing with a 1 mm inner diameter at a 5 mm depth and imaged at 808 nm (Vevo LAZR-X). The limit of detection (LOD) was set at 3*σ*/*k*. This calibration yields a probe concentration–PA relationship only; no Aβ-responsive PA signal was established.

### 2.5 Aβ1–42 preparation and conformational staging

The monomerization protocol follows that of Stine et al. (2011). Lyophilized Aβ1–42 was dissolved in ice-cold HFIP (1.0 mg mL^−1^), incubated for 1 h at room temperature, aliquoted (100 µg per tube), evaporated to a thin film over 12 h, and stored at −80 °C. Before use, each aliquot was redissolved in anhydrous DMSO (5 mM) and diluted to 50 µM in PBS pH 7.4 (DMSO < 2% v/v). The three conformational states were defined as monomers (0 h, 4 °C), oligomers (24 h, 4 °C, quiescent), and fibrils (72 h, 37 °C, 180 rpm). Quality was confirmed by ThT FL kinetics (LeVine, 1999) and TEM negative staining. Throughout this work, all Aβ1–42 concentrations—including those of the oligomer preparations—are expressed as monomer equivalent (total-peptide) concentrations, determined gravimetrically from the weighed lyophilized peptide and its molecular weight, and carried through the HFIP → DMSO → PBS dilution series. Because oligomers constitute a polydisperse ensemble without a defined molecular weight, no molar concentration of discrete oligomeric species is implied, and the aggregation state, rather than the concentration, was verified spectroscopically and by TEM. In all comparative assays, the different groups received the same monomer equivalent dose drawn from a common stock.

### 2.6 Aβ–nanoprobe interaction analysis

- Fluorescence quenching. FITC-Aβ1–42 monomer (5 µM in PBS) was titrated with Tf/KLVFF-PCN-222 (0–50 µg mL^−1^, 5 min equilibration per step; Hitachi F-7000, excitation 488 nm, emission 525 nm). The apparent Stern–Volmer relationship is given in Equation 2,
*F*_0_ and *F* are the fluorescence intensities of FITC-Aβ1–42 in the absence and presence of the probe, respectively; [Q] is the probe concentration (μg mL^−1^); and *K*_SV,app_ is the apparent Stern–Volmer quenching constant (mL μg^−1^).
$$\frac{F_{0}}{F}=1+K_{\text{SV},\text{app}}\;\left[Q\right],$$ (2)
  uses *K*_SV,app_ (mL µg^−1^) as a within-system relative parameter. Soret band inner filter effects were partially corrected using size- and ζ-potential-matched inert SiO_2_ nanoparticle blanks.
- ITC thermodynamics. Aβ1–42 monomer (10 μM, 280 µL in sample cell) was titrated with Tf/KLVFF-PCN-222 (normalized to 100 µM equivalent porphyrin) at 25 °C: one 0.4-µL preinjection followed by 19 × 2.0 µL injections at 180 s intervals, with 750 rpm stirring. Origin 7.0 with an independent binding sites model returned the apparent dissociation constant (*K*_d,app_), ΔH, ΔS, and stoichiometry n, all porphyrin equivalent normalized. The same normalization was used consistently across all ITC comparisons.
- CD. Mixtures of 12.5 µM Aβ and 12.5 µg mL^−1^ probe in PBS were co-incubated at 37 °C; CD spectra (0.1 cm path length, 190–260 nm, 50 nm min^−1^) were collected at 0, 6, 12, and 24 h using a probe-only baseline subtraction. The secondary structure was estimated with DichroWeb CDSSTR.
- Docking and MD (computational, mechanistic hypothesis only). Receptor structures PDB 1IYT (Crescenzi et al., 2002) and 2BEG (Lührs et al., 2005) were prepared using PyMOL 2.5/AutoDockTools 1.5.7. AutoDock Vina 1.2.3 (Trott and Olson, 2010) was used to run 20 replicates per ligand–receptor pair (exhaustiveness = 32); the three lowest-energy poses each entered three independent 50-ns AMBER 22 trajectories (ff14SB/GAFF2/TIP3P, 310 K NPT). Trajectory analyses covered RMSD, Rg, H-bond occupancy, and MM/GBSA relative binding energies. All outputs in Section 3.2 are framed as hypothetical binding modes supporting mechanistic discussion, not experimentally validated structural conclusions. Porphyrin Q-band assignments follow Gouterman’s four-orbital model (Gouterman, 1961) and the TD-DFT framework of Wamser and Ghosh (2022).

### 2.7 *In vitro* BBB model and cell experiments

hCMEC/D3 monolayer. According to Helms et al. (2016), collagen I/fibronectin-coated 12-well Transwell inserts (Corning 3460, PET, 0.4 µm) were seeded with hCMEC/D3 at 1.0 × 10^5^ cells cm^−2^, and experiments were conducted between days 7 and 10. Barrier integrity was confirmed by TEER and Lucifer yellow paracellular leakage (*P*_app_ < 5 × 10^−6^ cm s^−1^), with tight junction proteins (claudin-5, ZO-1, and occludin) verified by immunofluorescence. The TEER of this model (∼190 Ω·cm^2^) is substantially lower than *in vivo* BBB values (>1,500 Ω·cm^2^); therefore, *in vitro* *P*_app_ results should not be extrapolated to *in vivo* barrier-crossing efficiency.

#### 2.7.1 Transwell permeability

Four probe formulations (50 µg mL^−1^ each, in serum-free medium) were loaded apically; basolateral Zr content was measured by ICP–MS (Agilent 7900) at 0.5, 1, 2, 4, and 6 h. The apparent permeability coefficient was calculated using Equation 3:

*P*_app_ is the apparent permeability coefficient (cm s^−1^); d*Q*/d*t* is the steady-state rate of Zr appearance in the basolateral compartment (μg s^−1^); A is the surface area of the Transwell insert membrane (1.12 cm^2^); and C_0_ is the initial apical probe concentration (μg mL^1^).

$$P_{\text{app}}=\frac{dQ/dt}{A\cdot C_{0}}.$$ (3)

TEER was monitored throughout the experiment. Because ICP-MS cannot distinguish intact PCN-222 from released Zr species, *P*_app_ is formally an apparent Zr-element flux coefficient. Receptor competition. FITC-labeled Tf/KLVFF-PCN-222 was applied to hCMEC/D3 cells for 30 min, 2 h, and 4 h. Subsequently, EEA1 and LysoTracker Red colocalization was quantified by ImageJ JACoP from Leica TCS SP8 confocal z-stacks. Cells presaturated with free Holo-Tf (50-fold molar excess, 30 min) were reexposed to the FITC probe to assess the TfR1-sensitive flux fraction.

##### 2.7.1.1 Receptor competition and intracellular trafficking

To assess receptor competition and intracellular trafficking, FITC-labeled Tf/KLVFF-PCN-222 was applied to hCMEC/D3 cells for 30 min, 2 h, and 4 h.

#### 2.7.2 Neuroprotection

SH-SY5Y cells (1 × 10^4^/well) were treated with Aβ1–42 oligomers (5 µM final, monomer equivalent) that had been preincubated with a 50 µg mL^−1^ probe for 4 h. Viability was assessed by MTT (with porphyrin-MOF absorbance blank correction), membrane damage by LDH release (Roche), and Ca^2+^ dynamics by Fluo-4 AM confocal imaging. The tandem model placed hCMEC/D3 inserts above SH-SY5Y-seeded wells; following 6 h of apical loading and insert removal, basolateral viability was measured 24 h later. All results are bounded to this in vitro configuration.

#### 2.7.3 Statistics

Data are presented as the mean ± SD (n ≥ 3 biological replicates; exact n values are provided in the figure captions). A Student’s t-test was used for two-group comparisons, and a one-way ANOVA followed by a Tukey’s HSD was used for multiple groups (*p* < 0.05, GraphPad Prism 9.0). Curve fitting was performed using Origin 2022 (OriginLab).

## 3 Results and discussion

### 3.1 Structural characterization of PCN-222 and Tf/KLVFF-PCN-222

**FIGURE 1:**
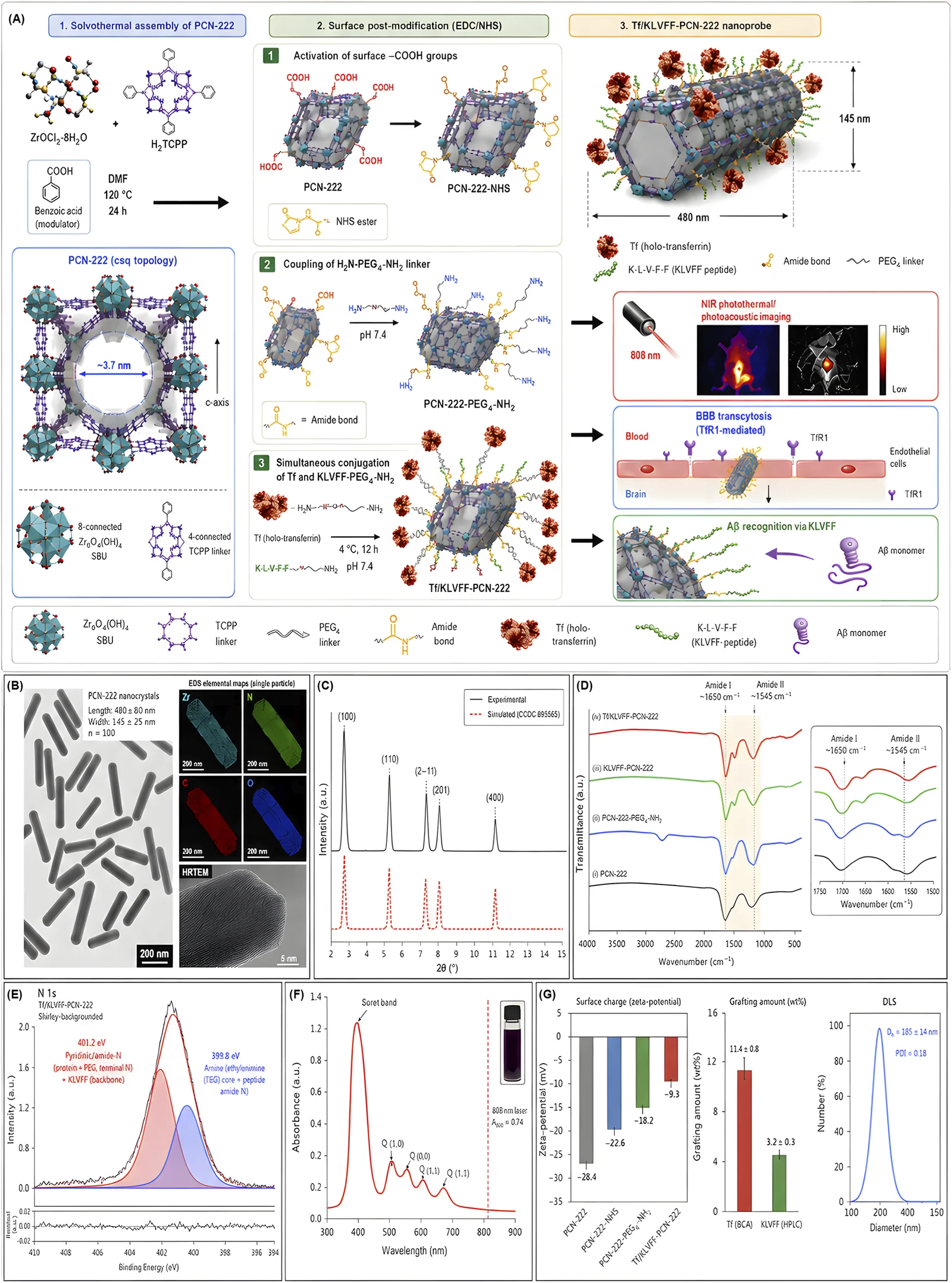
PCN-222 synthesis and structural characterization: **(A)** csq-topology assembly and dual-peptide conjugation schematic, **(B)** TEM morphology, **(C)** PXRD experimental versus CCDC 893545 simulation, **(D)** FT-IR spectra at each functionalization stage, **(E)** XPS N 1s high-resolution spectrum, **(F)** UV–VIS–NIR with Soret and Q-bands, and **(G)** zeta potential evolution and dual-peptide quantification.

Classical solvothermal synthesis gave PCN-222, onto which the Tf/KLVFF dual-peptide corona was subsequently built via a three-step EDC/NHS chemistry (Figure 1A). Read together, the structural data make a coherent case for both the csq phase purity and successful surface functionalization.

The diffraction pattern exhibited five characteristic peaks at 2θ = 2.5°, 4.7°, 6.6°, 7.1°, and 9.9°, indexed to (100), (110), (210), (201), and (400) reflections, respectively—matching the CCDC 893545 simulation in both position and relative intensity (Figure 1C; Feng et al., 2012), with no signatures from fcu-type PCN-224 or ftw-type MOF-525. Phase purity in this system is non-trivial: at least six Zr-TCPP polymorphs can emerge under similar conditions (Kafentzi et al., 2026; Morris et al., 2012; Park et al., 2016), and stoichiometric ratios alone offer no guarantee of phase selectivity. N_2_ sorption produced a type-IV isotherm with a BET area of 2,185 ± 45 m^2^ g^−1^ (n = 3), consistent with the reported benchmark (∼2,200 m^2^ g^−1^) (Feng et al., 2012). DFT pore-size analysis resolved two populations: a ∼1.3-nm micropore shoulder and a ∼3.7-nm mesopore corresponding to the csq one-dimensional hexagonal channel. This 3.7 nm aperture is wide enough for Aβ1–42 monomers (hydrodynamic radius ∼0.9 nm) and early oligomers to diffuse freely, whereas the UiO-66 fcu windows (0.6/1.1 nm) largely restrict this access. TEM images revealed uniform hexagonal rods (480 ± 80 nm × 145 ± 25 nm, n = 100), with EDS maps confirming the homogeneous distribution of Zr, N, C, and O (Figure 1B). ICP-OES determined the Zr content to be 18.7 ± 0.3 wt%, in close agreement with the csq theoretical value of 18.9 wt% (Supplementary Table S2).

After the three-step functionalization, FTIR revealed new features at 1,660 cm^−1^ (amide I) and 1,545 cm^−1^ (amide II) (Figure 1D). The XPS N 1s spectrum resolved two components at 399.8 and 401.2 eV (ratio ∼1:1.4), which is consistent with the mixed nitrogen environments from the KLVFF backbone amides, Tf protein nitrogen, and PEG4 terminal amines (Figure 1E). BCA assay returned a Tf loading of 11.4 ± 0.8 wt%; reverse-phase HPLC put KLVFF at 3.2 ± 0.3 wt% (measured Tf:KLVFF loading ratio ≈ 1:18 M, versus the 1:8 feed ratio used in conjugation—consistent with the more efficient grafting of the small peptide relative to the bulky protein), fitting the intended design of dense recognition peptide coverage with sparser protein anchors. Zeta potential stepped from −28.4 mV (bare PCN-222) through −18.2 mV (PEG4 intermediate) to −9.3 ± 0.9 mV for the final Tf/KLVFF-PCN-222. Hydrodynamic diameter after functionalization settled at 186 ± 14 nm (PDI = 0.18).

The UV–Vis–NIR spectrum displayed an intense Soret band at 420 nm alongside Q-bands at 515, 550, 590, and 645 nm (Figure 1F). According to Gouterman’s four-orbital model (Gouterman, 1961), the D2h symmetry of free-base porphyrin lifts degeneracy among the a1u/a2u → eg* π–π* transitions, splitting them into four resolvable Q-bands. TD-DFT on a truncated [Zr_6_O_4_(OH)_4_(HCOO)_8_ (TCPP)] model [B3LYP/6-31G (d,p)] predicts 519, 554, 589, and 648 nm (Wamser and Ghosh, 2022), deviating from the experiment by under 4 nm on average. Notably, 808 nm excitation falls in the low-energy tail of the Q-band rather than at the absorption maximum, sustaining a finite cross-section (A_808_ ≈ 0.74) while sidestepping the rapid photobleaching associated with full resonance.

The porphyrinic framework retains an intrinsic NIR photothermal response, which we characterize here as a material property rather than an Aβ-responsive readout. Under 808 nm irradiation (1.0 W cm^−2^), Tf/KLVFF-PCN-222 reached a photothermal conversion efficiency of η = 47.3 ± 1.8%, essentially matching bare PCN-222 (46.5% ± 1.5%) and confirming that dual-peptide functionalization leaves the porphyrin-core photophysics undisturbed; the probe was colloidally and optically stable over seven days and across five on–off irradiation cycles. In a tissue-mimicking phantom, the photoacoustic signal scaled linearly with probe concentration (probe PA-LOD = 1.6 µg mL^−1^). We emphasize that this is a probe concentration calibration only—no Aβ-responsive photoacoustic signal was established in this work. Full photothermal, colloidal-stability, and PA calibration data, together with a comparison against representative NIR contrast agents (Jiang et al., 2017; Wang S. et al., 2022), are provided in Supplementary Figure S1 and Supplementary Table S2.

### 3.2 Aβ recognition performance and mechanistic hypotheses

Four levels of evidence—apparent binding affinity, thermodynamics, secondary structure change, and computational prediction—are used to characterize how Tf/KLVFF-PCN-222 interacts with Aβ1–42. Six structural controls were concurrently evaluated to probe sequence specificity and topological contributions.

**FIGURE 2:**
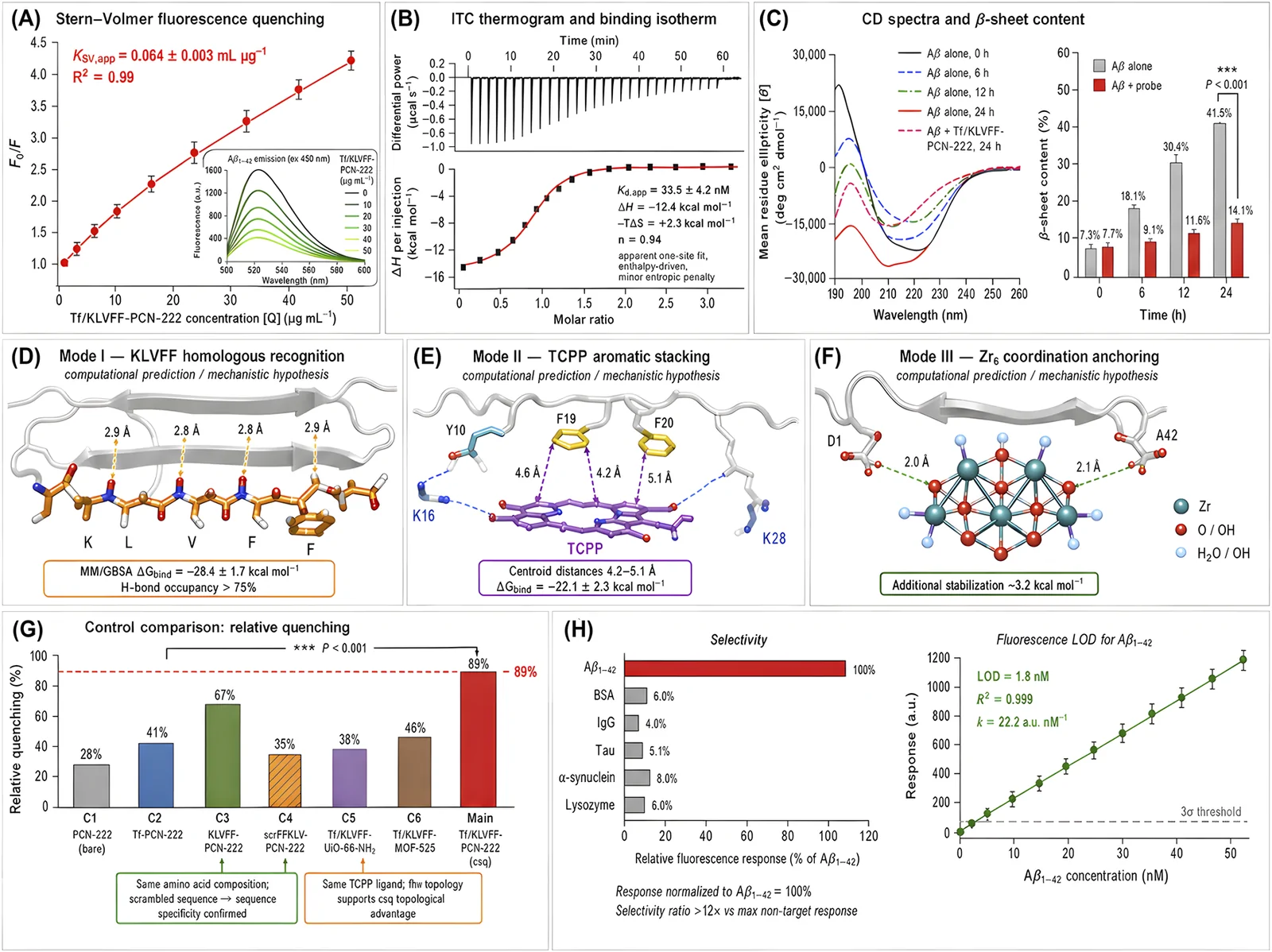
Amyloid-β (Aβ) recognition and mechanistic hypotheses: **(A)** Stern–Volmer quenching curves, **(B)** ITC isotherms and fitting, **(C)** CD spectra and β-sheet quantification, **(D–F)** hypothetical binding modes I–III, **(G)** structural control quenching comparison, and **(H)** selectivity profile and fluorescence (FL) limit of detection (LOD).

The Stern–Volmer analysis gave *K*_SV,app_ = 0.064 ± 0.003 mL µg^−1^, with *F*_0_/*F* rising approximately linearly across the probe concentration range (Figure 2A). ITC with the independent binding sites model returned *K*_d,app_ = 33.5 ± 4.2 nM, Δ*H*_app_ = −12.4 kcal mol^−1^, −*TΔS*_app_ = +2.3 kcal mol^−1^, and *n*_app_ = 0.94 (Figure 2B)—all porphyrin equivalent normalized apparent values. For context, Tf-PCN-222 and KLVFF-PCN-222 single-functional controls gave *K*_d,app_ values of 1.92 µM and 185 nM, respectively; co-presenting both Tf and KLVFF correlates with the strongest apparent affinity in this series.

CD analysis showed that the Aβ1–42 β-sheet content increased from 7% at 0 h to 41% at 24 h from 7% at 0 h to 41% at 24 h (incubated at 37 °C in PBS), reflecting spontaneous coil-to-sheet misfolding. In the presence of 12.5 µg mL^−1^ Tf/KLVFF-PCN-222, this value reached only 14% at 24 h (Figure 2C), suggesting that the probe substantially slows Aβ conformational conversion.

Docking and AMBER MD identified three hypothetical binding modes, each framed explicitly as a working hypothesis. Hypothetical mode I—KLVFF/Aβ16–20 homosequence recognition (Figure 2D): KLVFF and Aβ residues 16–20 may form an antiparallel β-strand pair, with four backbone H-bonds showing MD occupancy >75% and the lowest MM/GBSA relative binding energy (approximately −28 kcal mol^−1^). Hypothetical mode II—TCPP–Aβ aromatic π–π stacking (Figure 2E): the porphyrin macrocycle may engage Aβ residues F19, F20, and Y10 via π–π interactions (centroid–centroid distances 4.2–5.1 Å), potentially reinforced by electrostatic bridges between peripheral TCPP carboxylates and lysines K16/K28 (MM/GBSA approximately −22 kcal mol^−1^). Hypothetical mode III—Zr_6_ node Lewis acid–base anchoring (Figure 2F): residual µ_3_-OH/H_2_O groups on the Zr_6_O_4_(OH)_4_ nodes may interact with Aβ terminal carboxylates (D1, A42), providing supplementary stabilization; this remains speculative pending solid-state or solution spectroscopic validation.

The six structural controls at 25 µg mL^−1^ probe/5 µM FITC-Aβ gave relative quenching efficiencies of C1 bare PCN-222 (28%), C2 Tf-PCN-222 (41%), C3 KLVFF-PCN-222 (67%), C4 scrFFKLV-PCN-222 (35%), C5 Tf/KLVFF-UiO-66-NH_2_ (38%), and C6 Tf/KLVFF-MOF-525 (46%) versus 89% for the main probe (p < 0.001, ANOVA + Tukey, n = 4; Figure 2G). The scrambled FFKLV control C4 (35%) versus KLVFF (67%) supports the view that precise antiparallel β-strand pairing geometry drives recognition. C6 (MOF-525, ftw topology, same TCPP ligand) reached only 46% points toward csq mesopore geometry as a meaningful factor beyond TCPP identity alone.

Selectivity testing in a mixture of BSA, IgG, Tau, α-synuclein, lysozyme, and Aβ1–42 (5 µM each) confirmed the strongest FL response toward Aβ1–42, with a selectivity ratio exceeding 12 over the most reactive non-target (Figure 2H). The FL-mode apparent LOD was determined from the 3*σ*/k criterion (Equation 4),

σ is the standard deviation of the blank response (n = 10 independent blank measurements) and *k* is the slope of the linear fluorescence calibration curve.

$$\text{LOD}=\frac{3\sigma }{k},$$ (4)

was 1.8 nM—a useful benchmark for material–molecule interaction studies, although still above the picomolar to low-nanomolar range typical of clinical CSF/plasma Aβ42. Table 1 places this platform among representative Aβ recognition systems for context.

**TABLE 1:** Contextual comparison of Aβ recognition platforms.

Platform	Recognition/readout	Affinity/response	LOD or note	Mode	References
Graphene plasmonic platform	Aβ plasmonic shift	∼1,500 nM	25.0 nM	Plasmonic	Cui et al. (2021)
KLVFF-GO electrical sensor	Resistance versus Aβ42	—	Not comparable with FL LOD	Electrical	Scuderi et al. (2025)
KLVFF nanoassembly	Aβ capture/inhibition	—	Aggregation inhibition	FL/assembly	Wu et al. (2020)
Photo-triggered KLVFF nanofibers	Aβ aggregation modulation	—	Inhibition focus	Photoresponsive	Yang et al. (2018)
Cyclodextrin–KLVFF nanomagnets	Selective Aβ capture	Qualitative	Magnetic + MALDI-TOF	Magnetic	Mazzaglia et al. (2022)
Tf/KLVFF-PCN-222 (this work)	KLVFF + Tf + Zr_6_/TCPP	*K*_d,app_ = 33.5 ± 4.2 nM	FL LOD = 1.8 nM; PA-Aβ not established	FL; PA-capable	—

Aβ, amyloid-β; FL, fluorescence; GO, graphene oxide; *K*_d,app_, apparent dissociation constant; KLVFF, the Aβ16–20 pentapeptide Lys-Leu-Val-Phe-Phe; LOD, limit of detection; MALDI-TOF, matrix-assisted laser desorption/ionization time-of-flight; PA, photoacoustic; PCN, porous coordination network; TCPP, meso-tetrakis(4-carboxyphenyl)porphyrin; Tf, transferrin; Zr_6_, hexazirconium oxo cluster.

### 3.3 *In vitro* BBB apparent permeability and Tf competition sensitivity

**FIGURE 3:**
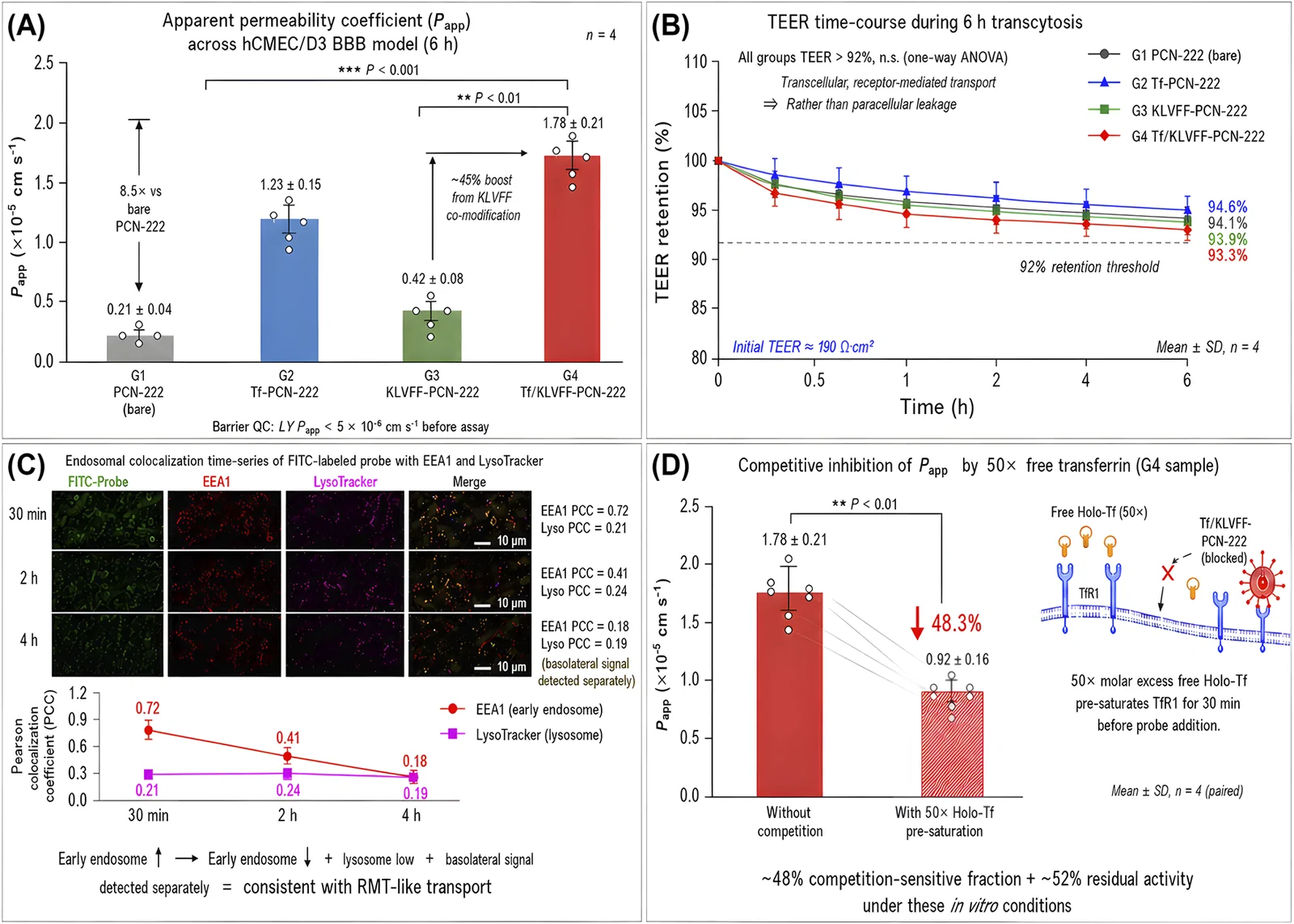
*In vitro* BBB permeability and transferrin (Tf) competition: **(A)** *P*_app_ for G1–G4, **(B)** TEER over 6 h, **(C)** colocalization time course with EEA1 and LysoTracker, and **(D)** *P*_app_ under 50× free Tf competition.

ICP-MS Zr tracking after 6 h placed the apparent permeability coefficients at G1 bare PCN-222, (0.21 ± 0.04) × 10^−5^; G2 Tf-PCN-222, (1.23 ± 0.15) × 10^−5^; G3 KLVFF-PCN-222, (0.42 ± 0.08) × 10^−5^; G4 Tf/KLVFF-PCN-222, (1.78 ± 0.21) × 10^−5^ cm s^−1^ (n = 4; G4 vs. G1: p < 0.001; G4 vs. G2: p < 0.01; Figure 3A). The increased flux tied to Tf decoration aligns with TfR1-mediated transcellular transport in this model (Johnsen et al., 2019; Lajoie and Shusta, 2015); the more modest gain from KLVFF co-modification likely reflects surface adsorption or non-specific endocytic contributions. TEER held above 92% throughout with no significant intergroup differences (Figure 3B).

The colocalization time course for FITC-Tf/KLVFF-PCN-222 with EEA1 and LysoTracker Red showed that at 30 min, the EEA1 PCC was 0.72 and the LysoTracker PCC was 0.21. By 2 h, EEA1 PCC fell to 0.41, while LysoTracker remained low. At 4 h, both PCC values had declined further, and Zr appeared basolaterally (Figure 3C). This pattern is directionally consistent with RMT-like transcytosis (Johnsen et al., 2019; Pulgar, 2019), although adsorptive or non-specific endocytic pathways cannot be ruled out. Presaturating TfR1 with a 50-fold excess of free Holo-Tf reduced the *P*_app_ of G4 from 1.78 × 10^−5^ to 0.92 × 10^−5^ cm s^−1^ (48.3% reduction; p < 0.01; Figure 3D). This indicates that approximately half of the apparent transcellular flux is TfR1 sensitive. All *P*_app_ values are formally apparent coefficients within the hCMEC/D3 model (Helms et al., 2016); direct basolateral particle integrity verification by AF4-MALS or TEM is deferred to subsequent works.

### 3.4 Mitigation of Aβ-mediated cytotoxicity and the tandem BBB–neuron model

**FIGURE 4:**
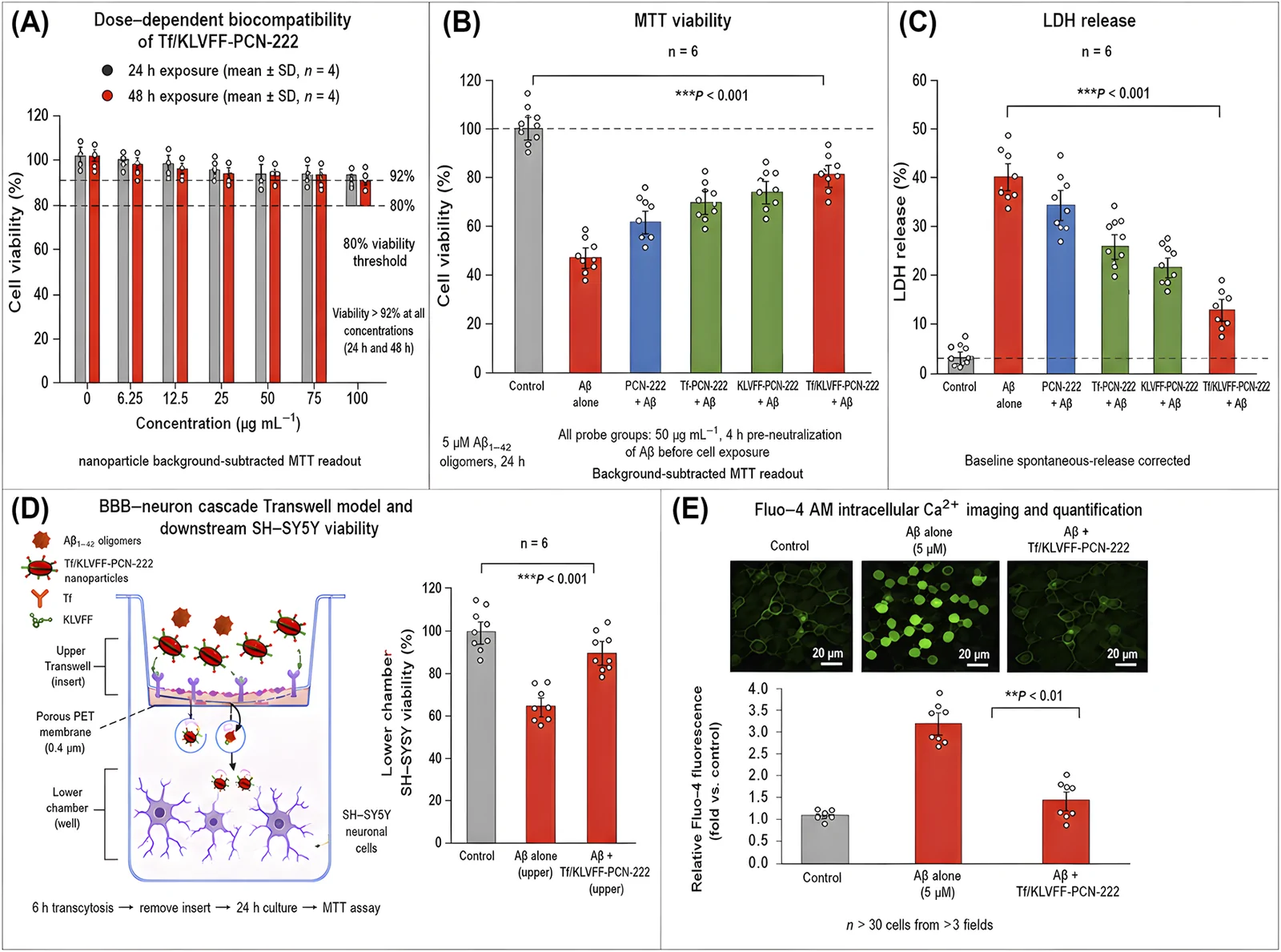
Amyloid-β (Aβ) toxicity mitigation and BBB–neuron tandem model: **(A)** dose–viability curve (MTT, blank corrected), **(B)** cell viability across six groups, **(C)** LDH release, **(D)** tandem Transwell schematic and basolateral SH-SY5Y viability, and **(E)** Fluo-4 AM Ca^2+^ imaging.

After correcting for porphyrin-MOF absorbance interference with the MTT chromogen, Tf/KLVFF-PCN-222 at concentrations up to 100 μg mL^−1^ maintained SH-SY5Y viability above 92% at both 24 and 48 h (Figure 4A). Adding 5 µM (monomer equivalent) Aβ1–42 oligomers without pretreatment decreased viability to 52% ± 6% and increased LDH release to 41% ± 5% (Figures 4B,C), which is consistent with established Aβ oligomer neurotoxicity (Howlett et al., 1995). Preneutralization with probe (50 µg mL^−1^, 4 h) before cellular addition improved viability across all probe groups: bare PCN-222 + Aβ (∼63%), Tf-PCN-222 + Aβ (∼71%), KLVFF-PCN-222 + Aβ (∼74%), and Tf/KLVFF-PCN-222 + Aβ reaching 85% ± 4% (LDH 13% ± 3%; n = 6; p < 0.001 vs. Aβ only). Fluo-4 AM imaging showed Ca^2+^-related fluorescence elevated in the Aβ-only group was partially attenuated by probe pretreatment (Figure 4E).

In the tandem hCMEC/D3-over-SH-SY5Y Transwell model, basolateral SH-SY5Y viability was measured 24 h later after 6 h apical loading and insert removal (Figure 4D). The Aβ-only apical group resulted in a basolateral viability of ∼68%, whereas the Tf/KLVFF-PCN-222 + Aβ group maintained viability at 92% ± 3% (n = 6; p < 0.001). These results are encouraging in an in vitro context; however, the substantial gap between this simplified system and in vivo physiology means they cannot be equated with neuroprotection in a living animal.

### 3.5 Structure–function summary and comparative discussion

Data from Sections 3.1 and 3.2—particularly the C5 (fcu UiO-66-NH_2_) and C6 (ftw MOF-525) controls—establish that csq-topology PCN-222 outperforms both a narrow-pore fcu Zr-MOF and a TCPP-based ftw Zr-MOF in apparent Aβ recognition under the conditions used here. The ∼3.7 nm csq mesopore channels are geometrically permissive to Aβ monomers and early oligomers in a way that UiO-66 (0.6/1.1 nm) and MOF-525 (∼1.7 nm ftw window) are not (Kafentzi et al., 2026), while high density TCPP display along the csq pore walls provides abundant, accessible recognition sites. Combining open mesopore geometry, dense porphyrin presentation, and NIR-I photophysical response within a single Zr-TCPP platform represents a structural compromise that the csq phase accommodates effectively. This reading is broadly consistent with the design principles reviewed by Kafentzi et al. (2026).

The ITC profile is enthalpy dominated (Δ*H*_app_ = −12.4 and −*TΔS*_app_ = +2.3 kcal mol^−1^), contrasting with the near-equal enthalpy–entropy compensation typically reported for free KLVFF–Aβ interactions (Tjernberg et al., 1996; 1997). Covalent tethering of KLVFF through a PEG4 linker plausibly preorganizes the peptide near its recognition geometry, reducing the conformational entropy cost of engagement, while the high local peptide density amplifies enthalpic contacts through multivalency. The microsecond MD work of Cobos-Montes et al. (2026), tracing KLVFF-driven stepwise detachment of Aβ monomers from oligomer termini, offers directional mechanistic support. Prior platforms—PCN-222@ICG@RVG (Wang J. et al., 2022), hybrid membrane-coated nanoparticles (Lin et al., 2024), and microglia-modulating micelles (Lu et al., 2022)—differ sufficiently in their targeting strategies and readout modes to resist direct comparison; broader overviews of MOF-based Aβ biosensing and AD nanomedicine are available in recent reviews (Chen et al., 2023; Cheng et al., 2021).

Two observations qualify this interpretation. The KLVFF-only control G3 (*P*_app_ = 0.42 × 10^−5^ cm s^−1^) exhibits a significantly lower apparent permeability than Tf-PCN-222 G2 (1.23 × 10^−5^ cm s^−1^), confirming that KLVFF is not a meaningful transcellular driver on its own. The incomplete TfR1 competition (48.3% suppression) leaves roughly half of the apparent transport through unidentified channels; caveolin-1 and clathrin pathway inhibitors, combined with dual-tracer experiments, will be required to resolve this. Five clear limitations apply: all functional data are derived from the hCMEC/D3/SH-SY5Y in vitro system (Helms et al., 2016); ICP-MS Zr tracking conflates intact particles with degradation products; computational binding modes are simplified-model hypotheses; the 1.8 nM FL LOD is a materials level figure above clinical Aβ concentrations; and the PA readout covers probe concentration calibration only.

## 4 Conclusion

The structural case for Tf/KLVFF-PCN-222 rests on a consistent body of evidence: PXRD, N_2_ sorption, TEM, and ICP-OES together confirm csq phase purity both before and after functionalization, and the open channel architecture together with the porphyrin-lined pore walls provides the structural basis for the recognition behavior reported here. Fluorescence quenching, ITC, CD, and molecular simulation—taken alongside KLVFF sequence specificity (C4) and topology controls (C5 and C6)—converge on Aβ1–42-selective binding and β-sheet suppression under the *in vitro* conditions examined here, with the quantitative binding and detection parameters detailed above. Consistent with these material properties, the csq framework preserves its NIR-I photothermal behavior after dual-peptide functionalization, a feature reported here at the materials level only and not coupled to any Aβ-responsive photoacoustic readout. Across the endothelial monolayer, dual functionalization raised the apparent transcellular Zr flux higher than that of any single-ligand control, and presaturating TfR1 with an excess of free transferrin suppressed approximately half of that flux. From a chemical–biology standpoint, the main contribution is an experimentally grounded structure–function dataset. Topology-controlled comparisons spanning fcu and ftw frameworks demonstrate the relative advantages of the csq phase for Aβ recognition while showing that open mesopore accessibility, dense TCPP display, and NIR-I photophysical capacity can coexist in one bioinorganic platform. Future directions include developing Aβ-responsive PA characterization at a fixed probe concentration; verifying transendothelial particle integrity by AF4-MALS or TEM; testing the platform in neurovascular-unit *in vitro* models and appropriate *in vivo* settings with ethics documentation; comparing PCN-222 with PCN-225 and NU-902 within the csq subfamily; and replacing KLVFF with recognition sequences for tau PHF6 or α-synuclein to evaluate broader applicability across amyloid-related diseases.

## Data Availability

The original contributions presented in the study are included in the article/Supplementary Material; further inquiries can be directed to the corresponding author.
