# Supplementary material for: Topology- and sequence-controlled β-amyloid recognition by a KLVFF/transferrin-functionalized csq porphyrinic zirconium MOF (PCN-222)
Source: Front Chem. 2026 Sep 9;14:1906393. doi: 10.3389/fchem.2026.1906393 (PMC13597743; doi:10.3389/fchem.2026.1906393)
Supplement: Supplementary file 1 [file DataSheet1.docx]

**Supplementary Material**

*Topology- and Sequence-Controlled β-Amyloid Recognition by a KLVFF/Transferrin-Functionalized csq Porphyrinic Zirconium MOF (PCN-222)*

Xiao Cheng and Hongen Wei*

* Correspondence: qkbq1926@outlook.com

**1. Additional Structural Characterization of PCN-222**

The morphological, textural and compositional parameters of PCN-222 reported in Section 3.1 and Figure 1 of the main manuscript are collated in Supplementary Table S1. Transmission electron microscopy (TEM; FEI Tecnai G2-F20, 200 kV) on 100 manually segmented nanocrystals gave a mean length of 480 ± 80 nm and width of 145 ± 25 nm (mean aspect ratio 3.3), with EDS elemental maps confirming homogeneous Zr, N, C and O distribution. N₂ adsorption–desorption analysis (Micromeritics ASAP 2020, 77 K, n = 3) yielded a Brunauer–Emmett–Teller (BET) surface area of 2185 ± 45 m² g⁻¹, a total pore volume of 1.42 ± 0.05 cm³ g⁻¹ and a mesopore fraction of 0.78 ± 0.02, with a DFT pore-size distribution centered at ∼3.7 nm consistent with the open csq channels. Inductively coupled plasma optical emission spectrometry (ICP-OES; Agilent 5800, n = 3) gave a Zr content of 18.7 ± 0.3 wt%, in close agreement with the csq-theoretical value of 18.9 wt% for PCN-222. Together these data corroborate the phase purity, permanent mesoporosity and framework composition of the material shown in Figure 1.

**Supplementary Table S1.** Morphological, textural and compositional parameters of PCN-222 from TEM, N₂ sorption (BET) and ICP-OES analyses.

| **Parameter** | **Method / Instrument** | **Value (mean ± SD)** |
| --- | --- | --- |
| Crystal length | TEM, FEI Tecnai G2-F20 (n = 100) | 480 ± 80 nm |
| Crystal width | TEM, FEI Tecnai G2-F20 (n = 100) | 145 ± 25 nm |
| Aspect ratio (L/W) | TEM, FEI Tecnai G2-F20 (n = 100) | 3.3 |
| Elemental distribution | TEM-EDS, 200 kV | Homogeneous Zr, N, C, O |
| BET surface area | N₂ sorption, Micromeritics ASAP 2020 (n = 3) | 2185 ± 45 m² g⁻¹ |
| Total pore volume | N₂ sorption, Micromeritics ASAP 2020 (n = 3) | 1.42 ± 0.05 cm³ g⁻¹ |
| Mesopore fraction | N₂ sorption, Micromeritics ASAP 2020 (n = 3) | 0.78 ± 0.02 |
| Dominant pore diameter | DFT model (N₂ sorption) | ∼3.7 nm |
| Zr content | ICP-OES, Agilent 5800 (n = 3) | 18.7 ± 0.3 wt% (theor. 18.9) |

**2. NIR Photophysical Performance and Probe PA Concentration Calibration**

The probe held up well across three physiologically relevant media over a week: stored at 4 °C in PBS, artificial CSF, and 10% FBS-PBS, the hydrodynamic diameter drifted less than 8%, PDI remained below 0.22, and Soret band intensity stayed above 95% (Supplementary Figure S1A).

Under 808 nm irradiation at 1.0 W cm⁻² for 600 s, a 200 µg mL⁻¹ suspension climbed from 25.0 °C to 57.8 ± 0.7 °C (ΔT = 32.8 °C), nearing thermal equilibrium around 480 s (Supplementary Figure S1B). The Roper one-dimensional cooling analysis gave η = 47.3 ± 1.8% (n = 3). Bare PCN-222 and Tf-PCN-222 controls measured 46.5 ± 1.5% and 45.9 ± 1.7%, respectively, indicating that dual-peptide modification left the porphyrin-core photothermal response essentially undisturbed. Supplementary Table S2 places this η among representative NIR contrast agents for magnitude orientation only.

Across five on/off cycles, ΔTmax varied by just 1.2% RSD and the Soret band retained 96.8% of its original intensity (Supplementary Figure S1D). In the tissue-mimicking phantom, PA signal tracked probe concentration linearly from 0 to 200 µg mL⁻¹:

$PA=0.0142 c+0.087 \left( R^{2}=0.991 \right)$	(S1)

yielding a probe photoacoustic limit of detection (PA-LOD) of 1.6 µg mL⁻¹ at 5 mm depth (Supplementary Figure S1C). At matched concentrations, ΔTmax and PA signal moved together (Pearson r = 0.992, p < 0.001), consistent with photothermal conversion as the dominant PA source. This LOD is a probe concentration figure and should not be confused with Aβ detectability; no Aβ-responsive PA signal was established in this work.

**Supplementary Figure S1.** NIR photophysical performance: (A) 7-day colloidal stability across three media; (B) photothermal heating curves and η values; (C) PA signal–concentration calibration in tissue-mimicking phantom; (D) Soret retention and ΔTmax over five irradiation cycles.

**3. Photothermal Conversion Efficiency Comparison**

**Supplementary Table S2.** Photothermal conversion efficiency (η) of Tf/KLVFF-PCN-222 and representative NIR PA/PT contrast agents.

| **η Contrast Agent** | **λex (nm)** | **η (%)** | **Comparison Note** | **Ref.** |
| --- | --- | --- | --- | --- |
| Gold nanorods | 808 | ~22 | Plasmonic reference; varies with aspect ratio and setup | Wang et al., 2022b |
| Indocyanine green | 808 | ~16 | Small-molecule NIR dye; limited photostability | Jiang et al., 2017 |
| Prussian blue nanoparticles | 808 | ~40 | Inorganic PA/PT benchmark; synthesis-dependent | Wang et al., 2022b |
| MoS2 nanosheets | 808 | ~28 | 2D transition-metal chalcogenide | Wang et al., 2022b |
| Nanographene–porphyrin hybrid | 1064 | 60–69 | High-efficiency 1064 nm agent; not directly comparable at 808 nm | Wang et al., 2022b |
| Tf/KLVFF-PCN-222 (this work) | 808 | 47.3 ± 1.8 | Probe PA-LOD = 1.6 µg mL⁻¹ under phantom conditions | — |

References (Wang et al., 2022b; Jiang et al., 2017) appear in full in the main manuscript reference list.
